# Peace Data Standard: A Practical and Theoretical Framework for Using Technology to Examine Intergroup Interactions

**DOI:** 10.3389/fpsyg.2018.00734

**Published:** 2018-05-28

**Authors:** Rosanna E. Guadagno, Mark Nelson, Laurence Lock Lee

**Affiliations:** ^1^Peace Innovation Lab at Stanford, Stanford University, Stanford, CA, United States; ^2^SWOOP Analytics, Sydney, NSW, Australia

**Keywords:** peace, Big Data, social media, research methods, data science

## Abstract

The current paper presents a theoretical framework for standardizing Peace Data as a means of understanding the conditions under which people’s technology use results in positive engagement and peace. Thus, the main point of our paper is that Big Data can be conceptualized in terms of its value to peace. We define peace as a set of positive, prosocial *behaviors* that maximize mutually beneficial positive outcomes resulting from interactions with others. To accomplish this goal, we present hypothetical and real-world, data driven examples that illustrate our thinking in this domain and present guidelines for how to identify, collect, utilize, and evaluate Peace Data generated during mediated interactions and further suggest that Peace Data has four primary components: group identity information, behavior data, longitudinal data, and metadata. This paper concludes with a call for participation in a Peace Data association and suggested for guidelines for how scholars and practitioners can identify Peace Data in their own domains. Ethical considerations and suggestions for future research are also discussed.

“People are people so why should it be, You and I should get along so awfully?”

–*Depeche Mode* (Gore, 1984)

## Introduction

Even the most casual perusal of the news headlines confirms what the classic 1980s new wave band *Depeche Mode* put so eloquently; we humans often focus on our differences, from largely visible social categories such as age, gender, race, ethnicity, language, and socio-economic status, to less visible characteristics such as sexual orientation, disability status, occupation, education level, religious affiliation, and political orientation (Brewer, 1991). Similarly, research findings in the psychological sciences support this notion. For instance, Henri Tajfel established that by randomly sorting people into meaningless groups (i.e., the “blue” vs. “green” groups commonly referred to as the minimal group paradigm), people begin to prefer their group over the outgroup. Thus, dividing the world into “us” vs. “them” is as automatic a process as blinking our eyes and has been shown to broadly affect people’s perception of others (Tajfel, 1970; Balliet et al., 2014). People automatically categorize others by visible social categories (i.e., gender and ethnicity), as part of this process (Brewer, 1988). On the Internet, these psychological processes are magnified by the one-to-many methods of communication typified by Web 2.0 technologies such as social media and review aggregating websites (Amichai-Hamburger, 2005, 2015).

While much research has demonstrated the many ways these group differences lead to conflict (e.g., Allport, 1954/1979; Sherif, 1961; Hogg, 2016), this paper takes a different approach. We suggest that the precise group differences that otherwise would cause conflict, can instead generate prosocial behavior and new wealth through structured *engagement episodes* – interactions mediated by various networked technologies (e.g., crowdsourcing applications, social media, texting, email). This is especially the case with services that facilitate person-to-person financial interactions, such as when people rent each other’s cars and homes, thereby creating new wealth, new relationships, and new opportunities to form friendships. We suggest that these services yield mutual benefit in excess of the cost of engagement for both interactants. It is these discrete episodes of engagement, and the mutually beneficial interactions they comprise that, we argue, constitute meaningful positive peace that can be measured in useful ways.

## Overview of the Present Paper

How do we observe and measure the amount of positive engagement generated by different actors and applications? We suggest that big data can be conceptualized in terms of its ability to understand and initiate peaceful interactions. Thus, our theoretical framework for Peace Data provides a starting point. This initial version of the Peace Data Standard generalizes, and applies to ANY mediating technology, from old-fashioned “landline” phone calls, or Internet-based financial transactions, to the latest connection anyone makes on crowdsourced social media or dating applications such as Snapchat or Tinder. In the present paper, we focus on these questions by presenting our theoretical perspective on Peace Data, presenting guidelines for the identification, collection, examples of, uses for, and value of Peace Data generated during mediated interactions. These guidelines make hypotheses about the four primary facets of Peace Data: group identity information, behavior data, longitudinal data, and metadata. We further present several examples, hypothetical and genuine, of contexts in which data consistent with our proposed Peace Data Standard could be collected. Next, we conclude with a call for participation in a Peace Data association, suggesting guidelines for how both scholars and practitioners can identify Peace Data in their own organizations and datasets. Finally, we present suggested directions for future research, and a conclude with a preliminary discussion of the many ethical considerations in the collection and use of Peace Data.

**FIGURE 1:**
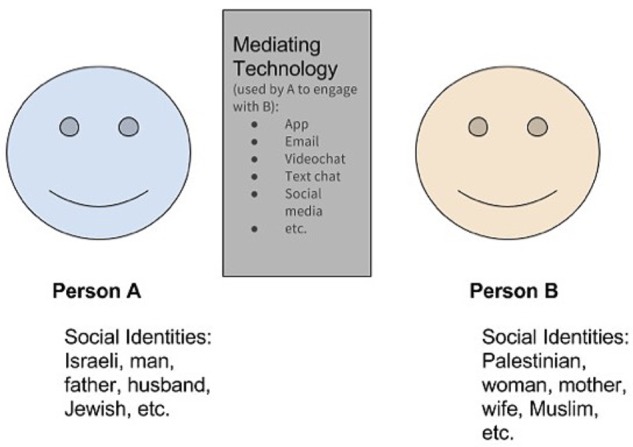
The primary dimensions of peace data.

*Mediating technologies*^1^, which we define as technology that “acts as an intervening agent, augmenting our ability to engage positively with others (Quihuis et al., 2015)” take on the role of a social actor (e.g., Reeves and Nass, 1996), connecting individuals acting independently by supported coordinated behavior. Thus, mediating technologies are those that connect people – oftentimes strangers – from divergent backgrounds to facilitate positive engagement. We define engagement in terms of both the quantity and quality of interaction. It can be either positive, reflecting high quality and frequent interaction or negative, defined as low quality and low frequency of interaction (see **Figure 1** for a visual of this process). Thus, these mediating technologies enable people to rapidly discover, refine, scale, and simultaneously assess in real-time the quantity and quality of mutually beneficial interactions between any two groups or entities. This in turn allows PeaceTech entrepreneurs, scholars, and designers to rapidly design, test, and validate interventions that effectively transform these group differences into raw material for sustainable peace (in which mutual benefit is equal to the cost of engagement) and eventually scalable positive peace (in which mutual benefit exceeds the cost of engagement). Prior to introduction of contemporary mediating technologies, the vast majority of human interaction was not easily recorded. In today’s world, we can easily record, analyze, and draw inferences – even in real-time – large samples of human interactions that occur via mediated-tech such as social media and mobile applications. We further suggest that these technological advances may even provide a means to remove resources and incentives from violent conflict situations, something we recommend as a direction for future research to explore.

### Peace Defined

Webster’s dictionary defines peace as: “a pact or agreement to end hostilities between those who have been at war or in a state of enmity” (Peace, n.d.). Cohrs et al. (2013) define peace as: “not only the absence or minimization of violence but also the presence or development of harmonious relationships (Anderson, 2004) and social justice (Galtung, 1969)” (p. 590). Furthermore, other research differentiates between negative peace – the more traditional perspective that pertains to the reduction, cessation, and prevention of violence – and positive peace – relief from violence and the introduction of social justice (e.g., Christie and Montiel, 2013). Building on these definitions, in our work at the Peace Innovation Lab at Stanford, we take both a behavioral and positive perspective, defining peace as a suite of positive, prosocial *behaviors* that maximize mutually beneficial positive outcomes from interactions with others.

## The Contact Hypothesis

In the present paper, we suggest that mediated-technology can be used to facilitate and measure peace, and specifically positive peace (that is, pro-social, and even mutually beneficial behavior across group boundaries). Given that it is widely established in psychology that, under the right conditions, contact between different groups can reduce intergroup conflict and facilitate positive interactions across group boundaries (Allport, 1954/1979), we suggest that if designed correctly, mediated-technology can increase positive peace. In support of this, research has shown that that mediated-contact, when properly designed and implemented can reduce intergroup conflict (Amichai-Hamburger et al., 2015; White et al., 2015). Specifically, both sets of the authors reviewed the results of several studies that illustrated the benefit of e-contact as an initial means of intergroup contact, particularly with respect to reducing intergroup bias and anxiety and increasing knowledge between groups. The authors theorized this is particularly effective when: e-contact takes place more than once at different time points, the interactants acknowledge both group similarities and differences, and the form of e-contact includes Allport’s (1954/1979) conditions for reducing intergroup conflict: equal status, common goals, cooperation, and support from authority). A Meta-analysis of over 500 studies on the contact hypothesis has verified its effectiveness of contact theory in its ability to reduce prejudice (Pettigrew and Tropp, 2006).

One theme apparent in both reviews is the notion that medicated-intergroup contact will be most successful when the interaction is structured in nature (Amichai-Hamburger et al., 2015; White et al., 2015). For instance, Amichai-Hamburger et al. (2015) defines structured contact as intergroup contact in which group members are selected for participation, the numbers are equal for each group, the contact is observed. Amichai-Hamburger et al. (2015) further suggest that mediated intergroup contact can be effective because of the following seven characteristics: “anonymity, control over the physical exposure, control over the interaction, ease of finding similar others, universal and constant availability and accessibility of the Internet, equality, and fun” (p. 517). Illustrative of this, the authors present examples of contemporary technology solutions built with the principles of contact theory. For instance, Games for Peace^2^ recruits Israeli and Palestinian children to interact in various virtual environments designed with the principles of contact theory to counteract negative stereotypes that people often hold of members of other groups and facilitate peaceful relations. Similarly, the authors also review a program called The Peace Factory^3^ which uses similar principles to foster peace in the Middle East by facilitating social media friendships between people from Middle Eastern countries in conflict. For instance, the Peace Factory launched a Facebook group called “Israel loves Iran”^4^ that provides a safe and public space for people from these two cultures to connect, communicate, and form friendships.

**FIGURE 2:**
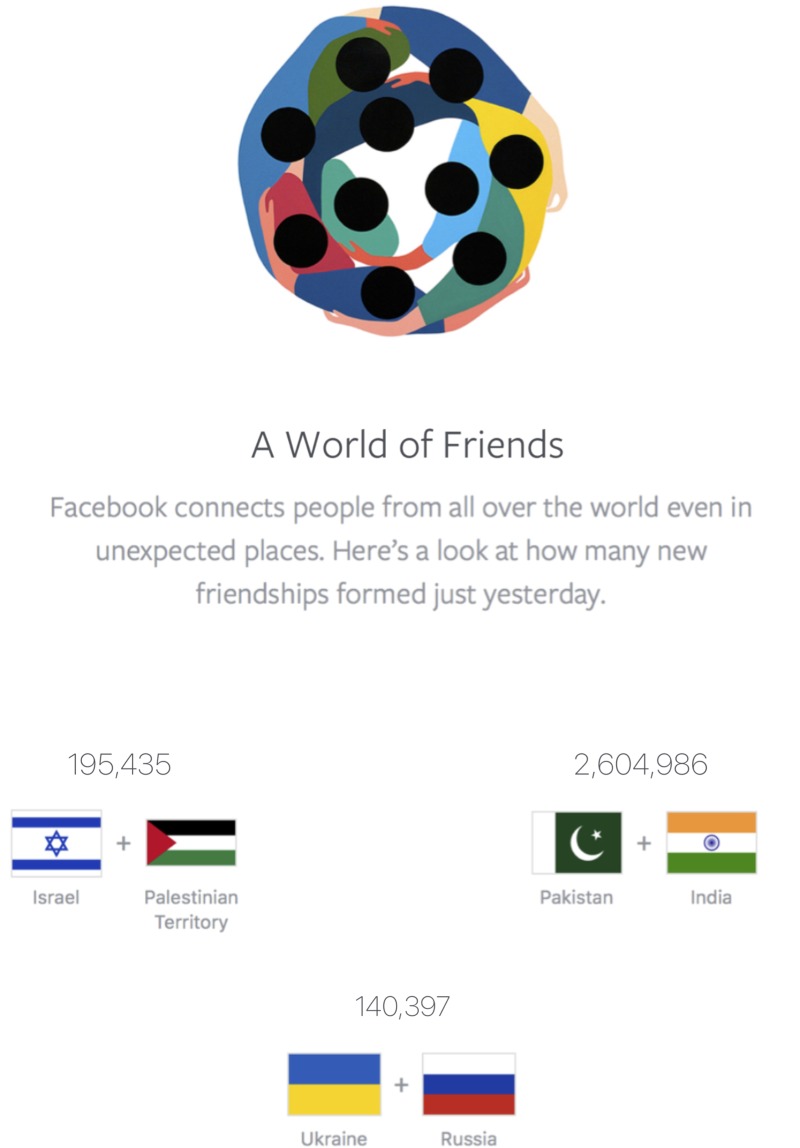
Screenshot of Peace Dot taken on February 19, 2018.

Our own lab also previously partnered with Facebook to create a page devoted to emphasizing the social media friendships created across conflict groups. Facebook’s Peace page (called Peace Dot^5^) reports the number of friend requests accepted between conflicting groups in real-time. It updates every 24 h and currently displays data on the friendships created across the following groups: Israel vs. Palestine, Pakistan vs. India, and Ukraine vs. Russia. This page also emphasizes the point that, even when conflict between these groups are high, people from these groups are forming more friendships than they are harming each other. For instance, Quihuis et al. (2015) reported that during a 2012 resurgence of Israeli-Palestinian violence, there were still over 13 times as many friendships formed on Facebook for each reported injury or death (see **Figure 2** for a screenshot of Peace Dot).

Other research has similarly found that technology can serve as an effective means of producing peaceful relations and reducing prejudice. For instance, Walther et al. (2015) placed Israeli and Palestinian students in a year-long intervention designed with the principles of contact theory. To examine this, groups of six students were placed in computer-based discussion groups as part of their participation in a course on Advanced Educational Environments. These students were members of three different in groups: secular Jewish, religious Jewish, and Arab (Muslim). The authors also recruited a control group that did not participate in the intervention. All participants filled out a series of pre- and post-measures of prejudice toward members of all three groups. Their results revealed that after communicating in their small groups throughout the entire academic year, participants were significantly less prejudiced toward the outgroups relative to both their pre-test scores and compared to the control group who did not participate in the intervention. Similarly, Cao and Lin (2017) reported that visual anonymity during interactions between people from different groups was effective in decreasing prejudice toward a specific outgroup member but was not effective in improving intergroup relations more broadly. However, when the authors added a video-based chat, they found that contact did improve attitudes toward different groups.

Taken together, the extant literature demonstrates the effectiveness of Internet communications technology designed using the principles of the contact hypothesis to facilitate peaceful interactions and reducing prejudice. However, this research focuses solely on the conditions under which communications technology can be used to improve intergroup relations but does not does not examine how technology can serve as a mediator of peace for face-to-face interactions. We suggest that in the today’s app-centric Internet, this is a more realistic use of technology in bridging the divide between groups. Indeed, Amichai-Hamburger and McKenna (2006) suggest that the use of technology as a mediator for conflict resolution occurs on a continuum that, if implemented correctly, can eventually transition into peaceful face-to-face interactions. Unfortunately, to date, little research has studied this transition. Given the paucity of research that studies the role of technology as a mediator of peaceful interactions in person, we provide hypothetical examples of the role mediating technologies play in facilitating positive, peaceful social interactions appear in **Table 1**. As both examples illustrate, ridesharing and the crowdsourcing of short- and long-term lodging by popular applications such as Airbnb, VRBO, Roomorama, HomeAway, etc. are both revolutionizing the way people travel and are also examples of the many mediating technologies that provide opportunities for positive engagement. Similarly, this paper was written collaboratively using a popular shared word processing software, and the widely used crowdsourcing platform Amazon Mechanical Turk provides similar positive engagement and new wealth between scientists in need of human research participants (e.g., Buhrmester et al., 2011) and between artists who crowdsource to design their art installations (burrough, 2016).

**Table 1:** Hypothetical examples of peace data.

Example 1. Ridesharing Applications	Example 2. Crowdsourced Lodging
On a recent trip to the airport, one of us (MN), used a popular ridesharing application on his mobile device to arrange his ride. Ride sharing applications (e.g., Gett, Uber, Juno, Lyft) have been gaining in popularity over traditional taxis, yet are not yet a widely accepted replacement for taxis (e.g., research reports that only 15% of American adults have used a ridesharing service, Smith, 2016). Despite this, growing anecdotal and scholarly evidence as well as an increasing market share all indicate that people prefer ridesharing to taxis (Deloitte Access Economics, 2016; Smith, 2016). In MN’s case, he spent most of the 42-min ride getting to know his driver, a young woman we shall call Ayanna^1^. Ayanna was a 19-year-old Muslim woman from Somalia who proudly wore a headscarf. As an older white man from Canada without a religious preference, MN seemingly had little in common with Ayanna. The two would not likely have ever encountered each other if not for the ridesharing application (also referred to as an App). As the ride progressed, MN asked whether Ayanna’s family approved of her job. Ayanna replied that her father and uncle were both taxi drivers out of a local airport and would never dream of letting her drive a taxi, not least because of the danger to her. However, with the ridesharing app tracking Ayanna’s location, her passengers’ identities and their driver-provided ratings, their pick up location, their intended destination, and the actual drop off location, the increased safety afforded by the technology made her family more than comfortable–they also thought this job would provide a great opportunity for her to help Americans personally experience how a black Muslim woman refugee from Somalia can be a valued and contributing member of society. So, for the cost of a $62 ride to the airport, the app enabled MN and Ayanna to discover each other, communicate both need and intent to meet that need, coordinate activity, trust each other even though they had never met, complete a mutually beneficial transaction, settle the transaction, and monetize the benefit. In the process, they created *and distributed* new economic, social, and arguably psychological wealth that could not have been generated without the mediating app. But unlike any other time in human history, all of these technology-enabled benefits are being passively measured and recorded*–in real-time*. The result? Over the course of 42 min, the ridesharing app enabled them to generate and record measurable positive engagement across ethnic, racial, religious, gender, nationality, language, and age boundaries–and measure some of the first-order economic and social impact of that engagement. Thus, this technology facilitated intergroup contact that was mutually beneficial for both parties, which as empirical evidence demonstrates, lead to a reduction in intergroup conflict (McKeown and Dixon, 2017).	When Pero and Gemma first learned about crowdsourced lodging, they decided to rent out part of their home as a way to supplement their income. Initially, that is exactly what happened. Not only did the couple increase their incomes, by sharing their home in this manner, they met people from all over the world, adding many of their guests to their circle of friends. Over time, as crowdsourced lodging became more popular through the use of various lodging-based social media sites (airbnb, vrbo, etc.), more and more of their neighbors started renting out all or part of their homes through these sites. Initially this was a boon to the local economy as more tourists came to visit the sights their European city had to offer. However, over time, Pero and Gemma started realizing that there were unintended consequences to their decision for their overall community. Local businesses run by their neighbors were among the first casualties of the share economy. First the local hardware store was replaced by a chain store that rents bicycles to tourists. This was followed by a number of local businesses being replaced by other tourist-centered (and expensive!) stores, restaurants, and services. Families who had lived in Pero and Gemma’s town for generations soon found that they could not afford housing in their city and many ended up moving to a nearby town without anything to draw in tourists. Thus, what started as a way to make some extra money and meet new people ended up disrupting the economy of local community and disrupted the bonds within the community as well. These unforeseen negative consequences of the share economy have led some to argue that crowdsourced lodging should be beneficial to the overall community not just the people renting out their property (van der Zee, 2016, October 6). Imagine Elia encounters Andres while seeking to rent his spare room for a weekend, through a home-sharing application. Andres’ posting of his spare room for rent is the initial *episode* of engagement between them, even though he has not met Elia yet and does not know who will reply, his willingness to engage is an important behavioral signal about both characteristics of his salient group identities, and about potential for engagement. Then Elia’s initial message to Andres about renting his room, gives us our first data point about the actual *relationship* not only between them, but also potentially between each of the groups Elia and Andres are members of. This includes both their broad, obvious group identities (e.g., men vs. women) and their more nuanced and previously much less visible group identities (e.g., Andres is a member of: Ph.Ds., retirees, atheists, Columbians, fathers of one, vs. Elia’s corresponding group identities of: 8th grade educations, working, Christians, Syrians, mothers of six). When Andres replies, perhaps about the dates the room is available, we can observe a second engagement episode, and these two episodes, one in each direction, create an *interaction.* Note that this is again an interaction both between them as individuals, *and* the groups they belong to (See **Figure 1**). Now, as we see a series of interactions between them over time, as they perhaps discuss price, amenities, and the duration of Elia’s stay, we can begin to quantify and model some precise qualities of their *relationship* that have never been visible before. Next, if we want to know more about the state of affairs between any two of the groups Elia and Andres are each a member of, we can aggregate all the relationship data, from other group members like them, to say something empirical about the *group dynamics* between, groups such as: Christians and Atheists, or Syrians and Colombians. Finally, depending on the interaction context, organizations may be able to attach an economic value to the interaction. In some cases, corporations may know this information, in others they may not; and when it is known, this value may change from before to after the interaction.

*^*1*^Note her real name but instead selected in homage to one of first author’s childhood friends.*

## Four Dimensions of Peace Data

As we conceptualize it, standardizing methods for the collection of Peace Data will enhance the ability of researchers, innovators, and practitioners to engage in an open and transparent examination of the effectiveness of mediating technology to promote peace and related outcomes. Below, we further expand on four psychological and methodological factor that should be considered as standard practices in Peace Data emerge. These topics are: group identity, the behavioral nature of Peace Data, the potential for longitudinal data collection, and the use of metadata as part of the Peace Data Standard. We further suggest that, while having all four dimensions represented in a dataset, having data which contains any one of these dimensions can be valuable in modeling the role of mediated communication in facilitating peace.

### Group Identity

Group identity data refers to the social categories people associate with themselves (e.g., “I am a student, a feminist, a parent,” Brewer, 1991), the groups that people sort others into both in terms of ingroup (e.g., “is that other person a member of my group?”; Tajfel and Turner, 1986) and in terms of the social categories that are easily observable (e.g., sex, ethnic background, age; Brewer, 1991). We hypothesize that new structural and statistical identities may also now be discoverable, through the analysis of big data using computer algorithms or machine learning. We further hypothesize that these identities may not discoverable by human observation and may instead emerge from consistency in one’s behavior. Illustrative of this, research indicates that certain technological solutions can: extrapolate people’s personality characteristics (i.e., extraversion) from the content and frequency of people’s online posts (e.g., Azucar et al., 2018), identify easily to persuade people from their online shopping behavior (Kooti et al., 2016), and develop profiles to differentiate customers into different groups based on their purchasing trends (Wen et al., 2018). People’s varying group identities are akin to *difference boundaries* – group difference categories that reflect a single or many, possibly nested differences in social identity (Leigh Star, 2010).

Note that, as the concealable stigma literature illustrates, not all social identities need to be known by both parties to affect a social interaction. Stigma refers to a social identity that is valued lower than other group identities (Crocker et al., 1998). Concealable stigmas are such undervalued social identities that can be hidden (Goffman, 2009). Identifying stigmatized social identities is somewhat context dependent and often results in discrimination against members of this group. Membership in a stigmatized group has been shown to negatively affect people’s physical and mental health (Major and O’Brien, 2005). Thus, people who are members of a stigmatized group (e.g., a chronic illness that is not readily observable) that can be concealed, may choose to do so to avoid these negative outcomes. Nonetheless, people with concealable stigmas carry the knowledge of their group membership with this knowledge can influence the expectations they carry into interactions, their responses to their interaction partners, and affect the impressions people for of them (Goffman, 2009). Related to the present paper, MacInnis and Hodson (2015) assert that mediated communication may provide a safe space for cross group relationships to forms. In their study, these examined how revealing a concealable stigma via mediated communication affected relationship formation between two interactants, one with a concealable stigma and one without. Their results indicated that revealing a stigmatized social identity early on in a relationship facilitates the formation of a stronger cross group relationship. Related to Peace Data, we hypothesize that there may be other, unknown or yet to be discovered social identities that may be revealed through the use of Big Data and Machine Learning to techniques to further understand how membership such a group affects the likelihood of an engagement episode resulting in an increase in peace for the interacts.

### Behavior Data

The second dimension of Peace Data is that it reflects people’s actual behavior. As much research indicates, directly recording people’s actions through *behavioral measures*, often provides unique insights about human social interaction (e.g., Baumeister et al., 2007; Lewandowski and Strohmetz, 2009). Prior to the era of big data driven by people’s increasing reliance on interactive, smart technologies, collecting meaningful samples of people’s behavior was considered too time consuming or labor intensive for many researchers to consider employing it in their research. Because increasingly ubiquitous sensors in our environment, with increasingly nuanced resolution and sensitivity, can now passively, unobtrusively, and automatically detect many kinds of human behavior, and especially human *social* behavior, the use of big data techniques to collect and analyze people’s behavior has led to new and novel insights about people. For instance, one study found that data on the frequency of different Internet search terms entered into Google were predictive of subsequent behavioral trends (Choi and Varian, 2012). Specifically, the researchers reported that this method could be used to predict seasonal variability in behaviors such as: visiting Hong Kong on vacation, unemployment trends (from searches for information on filing for unemployment insurance) and auto purchase trends (from search terms related to different types of cars). Applied to the question of Peace Data, we hypothesize that measuring actual behavior is essential for our understanding of the processes involved in producing and predicting peaceful interactions between people in from different groups.

### Longitudinal Data

A tertiary dimension of Peace Data is that it can be longitudinal in nature. Thus, scholars can use our model of peace to examine how prolonged positive engagement over time can facilitate peaceful outcomes between interactants. While this can be based on as little as a single engagement episode (e.g. clicking “like”) or social interaction (e.g., a Lyft ride) between two people, it can scale to multiple exchanges between the same people over time (e.g., renting the same vacation rental each year through the same crowdsourced lodging application) or can be applied to different people and different contexts over time. No matter how it is applied, we hypothesize that the longitudinal aspect of Peace Data presents an opportunity for people to understand how long-term positive engagement can facilitate peace.

Related to this is the question of how episodes of engagement between a pair of people affects others in their same situation or social system? Dynamical systems approaches to modeling human social behavior (e.g., Nowak et al., 2013) suggest that, over time, peaceful outcomes through positive engagement should spread throughout a social system and that it will spread fastest to the people most closely associated with the initial pair of interactants (e.g., Okdie et al., 2018, Unpublished).

### Metadata

The final aspect of Peace Data is that it can also include metadata, defined as data that provides information on other data (Ball, 2017). This can include aggregate data and/or descriptive statistics that provide group-level information on the mechanisms involved in achieving peaceful outcomes from social interactions mediated by technology. Metadata such as this allows scholars to track interactions across time and compare outcomes to other contexts, people, groups, and interactions. For instance, assessing the latency in messages sent between people through an app or social media site may reveal the extent to which people favor their own group members in a given setting of context. Currently the extant research on metadata in psychological science is scant. However, we hypothesize that, depending on the scope of the Peace Data collected, metadata can provide information about peace through positive engagement on different scales of measurement, for instance, at the level of an engagement episode or longer interaction and at the level of an individual pair or a larger group.

## Identifying Peace Data

How can scholars, innovators, and practitioners identify Peace Data? We suggest that any data that meets the criteria established in the four dimensions of Peace Data described is peace data. Consider this hypothetical interaction between two hypothetical people: person A and person B. Person A (Elsa, for the purposes of a generic example) has a variety of shared and unique group identities. For instance, she could be an African, Christian, mother of six. Person B (Toby, for the purposes of a generic example) may be a retired, atheist, Latino grandfather with a Ph.D. They may be from different countries, sexes, ethnicities, religious groups, and/or education levels (what we call *difference boundaries*; Leigh Star, 2010), but may then connect through some mediating technology. Difference boundaries can vary in many ways. For instance, a broad distinction between people is their gender^6^ (men vs. women). This distinction glosses over other group differences that may affect the outcome of the engagement episode. As the running example illustrates Elsa and Toby not only differ in gender, they also differ in religion, nationality, education, family size, and likely age. This raises concerns with respect to the generalizability of research findings to other people and engagement episodes and calls for guidelines for the ethical design of technologies that automatically detect and assign group identities to people.

## Peace Data Types

What kind of information do we recommend scholars, practitioners, and innovators collect in conjunction with Peace Data? To fully address this question, we must first review the relationship between people’s group identities, and how inter-group or cross-group-boundary engagement shapes their identities and affects their behavior. As indicated above, people have a tendency to divide the world into two groups: people who are part of their group, referred to as their “ingroup” vs. people who are not, referred to as an “outgroup” (Tajfel and Turner, 1986). The dividing line is typically based on salience – the social identity that is either most visible or most relevant to the context (Onorato and Turner, 2004). For instance, in a large group of men, two women with nothing else in common will likely prefer each other to other group members as gender is the salient social identity for that context. These same two women may feel anything but camaraderie when their nationality or political affiliations are the social identities more central to the social context. Research has shown that this is the case, particularly when interaction is mediated by technology (e.g., Spears et al., 2002).

**Table 2** shows examples of how Peace Data could be collected and formatted for data analysis. As sensor technology advances, we hypothesize that more precise detection, measurement, and modeling of prosocial, peaceful behavior in mediated interpersonal interactions will become possible. If enough people opt in to our proposed Peace Data Standard, we could follow a particular chain of engagement episodes between the same people, across platforms (e.g., eBay and PayPal). In this case the episodes are each visible on the respective platform, but the interaction is only visible across platforms.

**Table 2:** Example peace data formats.

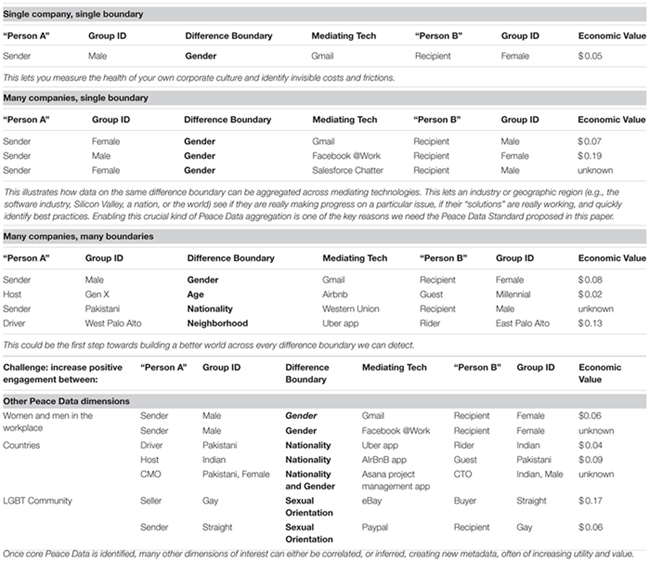

Additionally, ascertaining the level of analysis is also important to the collection of Peace Data. Since utility, value, and richness of the data likely increases longitudinally, we recommend the following process to determine the level of analysis. First, the point at which an *engagement episode* begins must be established (e.g., the first data point connecting A and B: when a male employee texts a female co-worker). Second, once an episode is reciprocated, it becomes an *interaction* (e.g., the female co-worker replies). Third, over time, a series of interactions accrue and can reveal novel, quantitative aspects of the *relationship* between A and B (e.g., A averages 39.5 min latency in response to messages from B). Fourth, an aggregation of relationships between others who share a group identity of interest with A and B (in this case other men and women in this or other workplaces), reveals previously invisible *group dynamics* about those groups of interest, and about the connections between them (e.g., finding that in company X, men typically take 45.75 min to respond to messages from female co-workers, whereas in company Y they take 32.5 min (resulting in an annual difference of $*x* in productivity, in which *x* can now potentially be quantified using internal corporate data^7^). These are the kinds of insights that can then inform effective interventions, research, and software design.

### A Social Network Analysis Example

We recently collected real world data consistent with our proposed Data Standard (Guadagno et al., 2018). Thus, while the two previous examples presented in **Table 1** were hypothetical in nature, this example comes from a social network analysis of data on employee’s social media use an Australian bank. In Western society, there is a commonly held stereotype that women talk more than do men. However, empirical investigations of this question have resulted in a different conclusion, one that suggests that the gender difference is in the opposite direction. For instance, the results of two meta-analyses that examined the question found that men generally talk more than do women (James and Drakich, 1993; Leaper and Ayres, 2007). Similarly, a study in which men and women’s daily conversations were recorded also concluded that the notion that women talk more than men is a negative stereotype with no real-world basis (Mehl et al., 2007). It is notable that these three research examples pertain to data that were collected long before the widespread adoption of Web 2.0 technologies such as social media.

With respect to social media use, myriad studies have demonstrated that men and women differ in the reasons they use the platform. For instance, research has shown that women generally use social media for relationship maintenance and social comparison purposes, while men use social media to make new connections (Haferkamp et al., 2012; Muscanell and Guadagno, 2012). What we currently do not know is whether men and women’s networking patterns change when the context changes to the workplace; and especially the larger corporate environments, that have been traditionally dominated by male executives, in command and control work structures.

**FIGURE 3:**
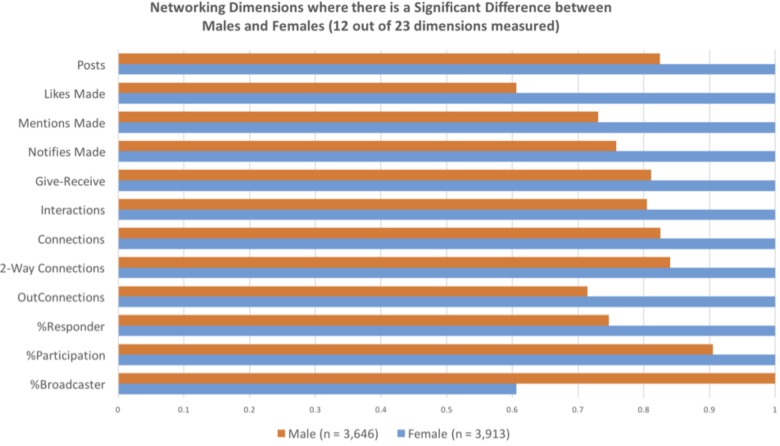
This graph displays standardized gender differences for the 12 communication dimensions in which women outperformed men. These bars compare the relative, standardized gender difference on the communication dimensions in the Guadagno et al.’s (2018) social network analysis. Maxed out bars indicate the group that significantly outperformed the other group.

**FIGURE 4:**
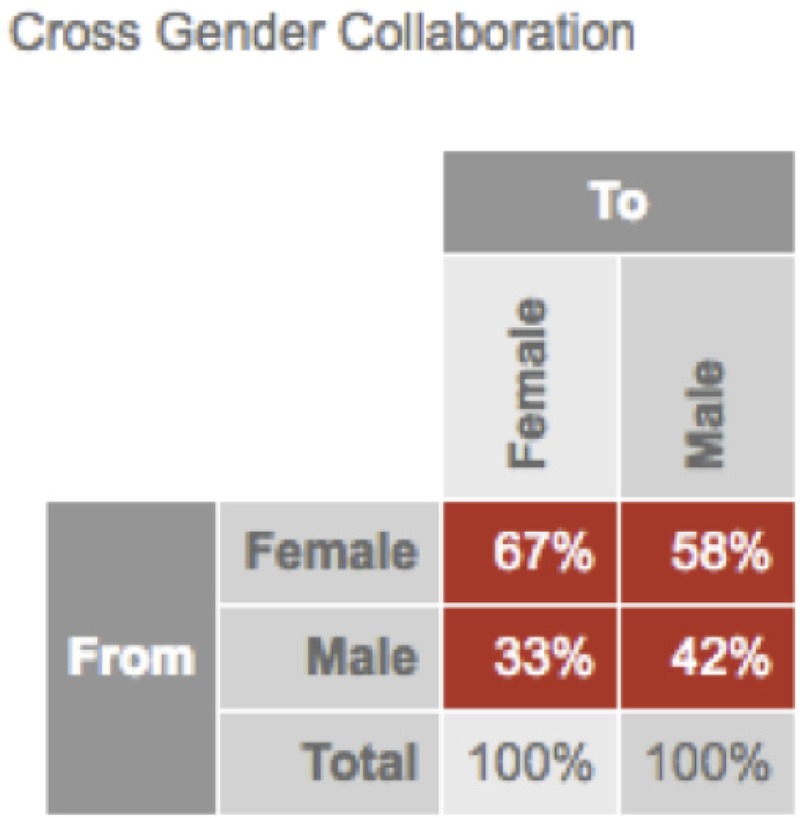
Cross-gender collaboration. This image indicates the percentage of participants by gender in Guadagno et al.’s (2018) social network analysis who sent messages to men vs. women.

To test the question of gender differences in the use of social media, the researchers measured the online social networking patterns of a large financial institution over a period of 6 months. Anonymized data from the online collaboration platform (Yammer – a Microsoft-owned social media platform for the workplace^8^) tracked participants contributions overtime. The only group identification collected was participant gender. Data were collected from over 7,500 employees with an approximately 50/50 gender split. The results of this research were stark: Women were more collaborative and communicative networkers than men, on most dimensions. For instance, across 23 collaborative measures (see **Table 3**), 12 showed a statistically significant difference between men and women in their technology-mediated social interactions at work (see **Figure 3**). As the results indicate, the women lead on all dimensions with a significant gender difference. However, it should be noted that one of the dimensions – %Broadcaster – a higher score is interpreted as having a negative connotation owing to the lack of reciprocal interaction reflected in a high score, and therefore provides the only dimension that men outperform women (Guadagno et al., 2018). **Table 3** provides a brief description of the dimensions used and **Figure 3** displays the standardized gender difference for each dimension on which men and women differed. Our analyses also revealed that women reach out to male colleagues far more often (58%) than men reach out to women (33%) co-workers (see **Figure 4**).

**Table 3:** Yammer communication dimensions.

Dimension	Description
Posts^∗^	Average number of posts made
Replies Made	Average number of replies made
Replies Received	Average number of replies received
Likes Made^∗^	Average number of likes made
Likes Received	Average number of likes received
Mentions Made^∗^	Average number of mentions made
Mentions Received	Average number of mentions received
Notifies Made^∗^	Average number of notifications made
Notifies Received	Average number of notifications received
Give Receive^∗^	Balance of Giving (outward) minus Receiving (inward)
Interactions^∗^	Average number of total interactions
Connections^∗^	Average number of unique connections
2-Way Connections^∗^	Average number of two-way connections
Replies/Post	Average number of replies received for each post
Reciprocity	Proportion of connections that are two-way (reciprocated)
In-Connections	Average number of inward connections (e.g., people who have replied to you)
Out-Connections	Average number of outward connections (e.g., people who you have replied to)
Diversity	Average breadth of Yammer groups actively participated in
%Participation^∗^	Average % of those active more than once every 2 weeks (non-observers)
%Engager	Average % Engagers (have a balance between giving and receiving)
%Catalyst	Average %Catalysts (receive more than they give)
%Responder^∗^	Average %Responder (give more than they receive)
%Broadcaster^∗^	Average %Broadcaster (post more but receive less responses)

*The variables with asterisks next to them indicate that women were significantly higher than men on that particular dimension*.

**FIGURE 5:**
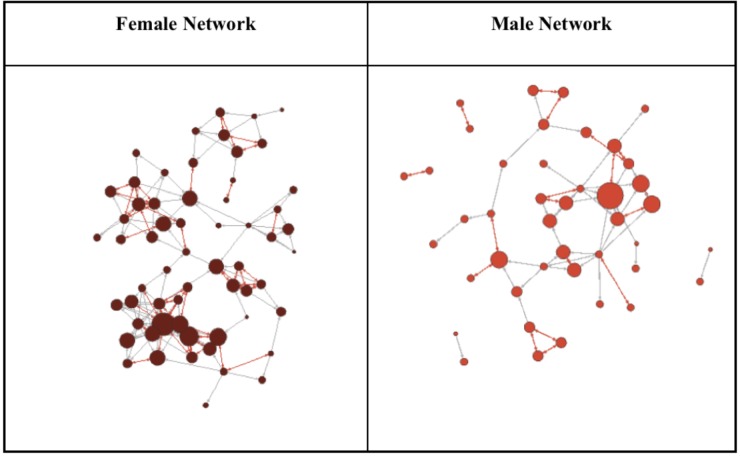
The following diagram compares the female only network with the male only network from Guadagno et al.’s (2018) social network analysis example. The red lines show reciprocated relationships, the gray lines show relationships, and the red dots indicate network nodes with larger nodes indicative of more connections and therefore more influence with the communication network.

Next, we examined the communication that occurs within and across difference boundaries. These results also revealed that women had denser and more reciprocal communication networks relative to their male colleagues (see **Figure 5**). Thus, while the data reported by Guadagno et al. (2018) clearly demonstrates meaningful differences in behavior across the difference boundary of gender. As discussed below, these results can contribute to our understanding of the role of mediated communication in facilitating prosocial behavior.

The Australian banking data uses gender as the relevant group identity (Guadagno et al., 2018) and we suggest that this aspect of our data illustrates the first dimension of Peace Data. One potential extension of this research would be to look for subgroups within gender. For instance, we could compare participants from the Australian banking study that did not differentiate between men and women in their messaging behavior from those that did as a first step in understanding what aspects of group identity (in addition to gender) determine who people are likely to message. Furthermore, illustrative of the second dimension of Peace Data, our Australian banking data example uses the actual behavior of men and women in the organization to assess the extent to which men and women communicate to others in their workplace, with women engaging in far more communication relative to men. Similarly, given the longitudinal nature of this data, it is also illustrative of the third dimension of Peace Data. Finally, a great deal of metadata – our fourth dimension of Peace Data – can be extrapolated and analyzed from underlying data; for example, message timestamp *data* in our Australian bank example above allows the calculation of response latency *metadata*. This means much progress can be made by discovering novel metadata calculation formulas (Guadagno et al., 2018). The reciprocal aspect of the data was determined from metadata recorded along with the content of the messages. Finally, what is unknown with this data is the emotional valence of the message content and how this plus the greater communicativeness of women over men relate to economic outcomes such as salary and promotions.

## General Discussion

Mediating technologies, like many technologies, are a double-edged sword owing largely to the unanticipated positive and negative consequences of technology. While they can connect geographically distributed people from all facets of people’s lives, much emphasis has been placed on the downsides of this connectedness (e.g., negative social comparison, viral spread of disinformation, divisive, uncivil discourse; Wiederhold, 2016). Since many of these negative effects are likely to be pursued by bad actors (such as trolls, social engineers, hackers) no matter what, we suggest that these same technologies need to also be deliberately designed, used, and cultivated to increase peace, by measurably increasing positive engagement across difference boundaries. Our framework can be applied to a wide variety of issues that organizations and the world at large are currently grappling with, such as increasing inclusivity for women and other members of underrepresented groups in the software industry and the military. As much research demonstrates, diversity of thought and perspective enhances innovation (Nielsen et al., 2017), therefore facilitating positive engagement between people from traditionally underrepresented groups and people in the traditionally dominant group for an occupational category (e.g., software engineer, air force officer) can enhance innovation and success in organizations.

The research and hypothetical examples presented in our paper illustrate how we can now start to identify a broader variety of group identities, some of which may have been unknown before the era of big data allowed using people’s digital footprints (e.g., Kosinski et al., 2016). Group identity data may also vary. As previous research has demonstrated, people have many different social or group identities, and the importance of each on people’s behavior is context dependent (e.g., Turner et al., 1994). Thus, the nature of the interaction, the social identities of the others present during the interaction, the setting in which the interaction takes place, all affect the outcomes, and, in the age of Big Data, we suggest that all of these features should be recorded and considered with Peace Data. Finally, in addition to the new, emerging group identities, there is also the question of the group identities created (often though not always unintentionally) by the people/cases left behind as these new kinds of groups are identified and categorized (Leigh Star, 2010). These *residual categories* are the ones that are discarded as unimportant by the data analyst. Leigh Star (2010) argues that determining who the residual categories are and how they are identified in datasets has important ethical implications for understanding certain instances of people’s actions and behaviors.

### Peace Data Uses

Why collect Peace Data? As illustrated throughout this paper, we argue that Peace Data is useful for many purposes. For instance, Peace Data can reveal largely unseen, invisible relationships and dynamics within those relationships. This can allow use to precisely measure and predict peace akin to Google Earth for social interaction. Furthermore, a fruitful future direction involves the use of Peace Data to determine the economic value of peace (i.e., what is the economic value of a certain action? For instance, it may be that a bank teller warmly greeting a customer is worth 3.17 cents to banks by maintaining a warm friendly atmosphere in a neighborhood where people know each other by name. While the economic value would vary by context (e.g., differ by community and neighborhood, by time of day), and across the additional difference boundaries such as race, gender, and profession. Thus, it may be possible to determine whether the data itself has economic value in addition to the positive prosocial behavior it signifies; for instance, hypothetically, we could use Peace Data to determine that a warm greeting in a high-crime neighborhood at 2:15 a.m. may be worth $4.33, while a similar warm greeting in a lower-crime neighborhood at 1 p.m. may only be worth $0.79. The value of having this Peace Data becomes clearer when it is applied to inform designers and engineers of peace technology interventions. Furthermore, we assert that insights gained from Peace Data can also be useful to communities seeking to improve the quality of life, health, and social capital. Finally, we suggest that this knowledge may have unintended negative consequences such as driving away consumers, businesses, and residents in geographic locations which generate less peace-related outcomes. Thus, the ethics regarding both modeling and reporting about the economic Peace Data should be carefully considered to avoid such unanticipated negative outcomes.

### Ethical Considerations

The proposed research agenda presented in this manuscript is not without serious ethical concerns pertaining to participant consent, security of participant data, and participant anonymity. Consistent with this, Gosling and Mason (2015) noted that one of the ethical issues associated to the study of mediated communication is that: “researchers have less control over and knowledge of the research environment and cannot monitor the experience of participants, or indeed their true identities, raise a number of ethical issues (Buchanan and Williams, 2010)” (p. 894). Thus, we recommend that companies and scholars interested in adopting our proposed Peace Data Standard adopt practices the protect their participants, particularly when they are customers as well.

With respect to participant anonymity, Chandler and Shapiro (2016) point to issues concerning participant anonymity as one of the main challenges of this area of research. For instance, Dawson (2014) provided evidence outside parties can sometimes identify participants whose text-responses were collected via networked applications. If an interested party has the right expertise and/or uses data triangulation methods such as a Google search, the author provided evidence that it is possible to identify participants despite the anonymization of the data. Specifically, Dawson (2014) was able to identify the source of a text passage directly quoted in 10 of 112 articles identified as relevant to this study. Furthermore, of these 10 articles containing identifiable data, the authors of five articles neither anonymized the text nor discussed ethical considerations, and the authors of one article tried unsuccessfully to anonymize the data. Thus, while it is important to safeguard participants’ anonymity and confidentiality, more may be required in terms of data obfuscation to protect people’s data.

Gosling and Mason (2015) further note that many of the ethical challenges of collecting data from people’s networked application use has arisen from a lack of guidance from ethics organizations because the rules developed for the ethical use of human research participants were developed before research on the Internet became ubiquitous. With respect to participant privacy, these authors suggested the criteria for defining behavior as “public” and therefore open for use in research without participant consent, should be carefully considered. They assert that this of particular concern when researchers, both in industry and academia, use web scraping techniques to collect data from Internet-comment and discussion forums such as social media, blogs, and other online discussions.

Similarly, as people’s online behavior grows and changes over time, people unintentionally build digital dossiers – a file or set of files containing detailed records about a person’s activities on the Internet – on themselves based their Internet use (Guadagno, in press). This accumulation of data has been further enhanced by services provided by social media companies (e.g., Facebook and LinkedIn) that provide the option for people to use their social media user ID and password in to access third party websites. This allows people’s online activities to be tracked across different Internet venues and provide useful data on people’s technology use, but it comes at a cost to people’s online privacy. While we suggest above that rich and detailed Peace Data could be collected by tracking people’s application use across websites and services, this should not occur at the cost of participants’ privacy and anonymity. As far as we can ascertain, there are no current widely adopted guidelines among researchers on this issue. However, we recommend the insightful and detailed questions to consider proposed by Buchanan and Williams (2010) pertaining to ethical considerations such as how to determine whether a specific Internet behavior is “public” and can be recorded and/or observed without consent. Finally, while many corporate entities fold blanket consent to participate in research as part of end user licensing agreements, we further suggest that industry researchers consider crafting more direct and more educational consent procedures to both inform and protect the people whose data they collect.

### Limitations and Future Directions for Research

We are in the nascence of technological growth especially with applications that leverage large scale group dynamics inherent online (e.g., crowdsourcing applications, review aggregators, social media). While there are obvious unintended negative consequences (i.e., fake news spreads rapidly unchecked, the divisive direction of online discussions often take, especially if the topic is controversial) of this rapid technology growth, in this paper, we questioned if it is possible to turn this rapid technological growth into something positive? The Peace Data Standard presented in this paper is a first step in leveraging the power of Big Data and machine learning to start to understand when and how technology facilitates peace and positivity – people being good to each other. This paper is written as a conceptual paper and, as such, we did not include a discussion of the different database, programming, and data analysis tools that are currently popular with people who work with big data (e.g., R, Python, JavaScript, and SQL) as these tools may change over time.

The Peace Data Standard presented in this manuscript suggests several clear future directions for research. First, we can begin to identify different subgroups with increasing resolution and precision, particularly based on behavior sequences (which we refer to as engagement episodes); categorizing and correlating prosocial behaviors with increased precision. This correlation between behaviors and/or sequences of behaviors to outcomes of interest will generally occur longitudinally. Furthermore, we suggest that scholars in industry and academia with backgrounds in fields such as behavioral economics take up our call to build statistical models to understand the economic benefits of Peace Data. Similarly identifying the kinds of additional metadata useful to predict outcomes related to understanding the role of technology in facilitating peace is also an important for understanding the underlying mechanisms that promote positive engagement episodes resulting in peaceful outcomes.

Additionally, while many corporate entities likely already examine this internally, we would like to call for future research to examine the economic aspects of the different behaviors peace-relevant we can track using Big Data. Finally, given the paucity of research on the interplay between relationships that form online then transition to offline contexts, we would like to make a call for more research on the ways in which contact initiated online and/or used in context with offline interactions affects interpersonal processes.

### An Invitation

In conclusion, we see many opportunities for contribution from experts across many disciplines:

1. *Help improve the standard.* We very intentionally propose this as the version 0.1 beta of the Peace Data Standard. It should be dynamic, and there is much room for improvement from many intersecting domains. We therefore invite colleagues from academia, industry, and government around the world to respond to this paper with recommendations for further refinements for future release versions of the Peace Data Standard. These refinements should reflect, but not be limited to, psychological, sociological, technological, ethical, and methodological advancements in data collection and analysis from large-scale, real-time mediated technology. This includes taking advantage of increasing capability in the underlying sensor and computation technologies that make Peace Data collection and analysis possible. We propose working together toward establishing version 1.0 as a formal ISO standard for Peace Data. This is vital to enabling the kind of comparative analysis across platforms, organizations, communities, and locations, that will yield the fastest and cheapest discovery of what works best, where, when, and under what conditions, thus providing the greatest benefit for all.
2. *Help source Peace Data.* Colleagues in academia can teach students to help local businesses identify and submit their Peace Data. Colleagues in industry can spread the word internally and in their sector about the value of a company’s Peace Data, for things like regulator relations, customer engagement and loyalty, talent recruitment and retention, and more.
3. *Help make Peace Data auditable, so it becomes an effective market signal for (at least some of) the true value peace creates.* Organizations with Peace Data must be able to guarantee the validity of their data to preserve its value, and organizations and individuals relying on that data must be able to ensure it has not been tampered with or inflated. Therefore, third party audit standards and processes need to be developed jointly by all stakeholders, to ensure reliability and preserve meaning. Given the huge scale, granularity, and real-time nature of Peace Data, there is also a need for these audit processes to be automated as much as possible.

We further invite readers to consider whether this framework for Peace Data is applicable to the type of data collected in their research and by their organizations. While it is becoming more common in industry to hire data scientists (De Mauro et al., 2017), we suggest that data scientists can be particularly helpful in employing our proposed Peace Data Standard. Specifically, data scientists can be enlisted to help identify data that could be used to promote peaceful interactions through the use of mediated technology, sitting in the epicenter of Silicon Valley we understand firsthand the scarcity of good data scientists.

4. *Help establish a Peace Data Prize.* Having an established Peace Data Standard will enable companies who generate Peace Data to show their audited peace impact to regulators, employees, and customers. The public relations benefit alone, not to mention the improved customer loyalty and engagement, or the talent recruitment and retention benefits, may enable member companies who submit their Peace Data for audit to pool a small percentage of that aggregate new value, and fund a series of Peace Data Prizes, which the Peace Innovation Lab (PIL) at Stanford^9^ will award annually to customers, employees, and companies who have had the greatest per capita peace impact, who have made the greatest peace improvements, and so forth. This same arrangement can be repeated for municipalities, national governments, civil and religious organizations, and so forth. We propose to also set aside a portion of these funds for research, and for prizes for the best research using Peace Data each year. Interested organizations, corporate or government, should contact the PIL to register their participation.

### Implications

For industry, this Peace Data Standard facilitates a results-based economic mechanism for stakeholders to invest directly in peace. We argue that this enables a new kind of precision peace, in scenarios such as follows: first, from the data we presented above, any company wishing to understand gender differences in workplace behavior, can now measure within- and cross-gender engagement in the workplace, to identify whether there are any problems with gender discrimination, and, if so, what interventions actually work in their context. Second, for a US bank that needs to meet their Community Reinvestment Act (CRA) compliance requirements, being able to post a results-based contract for any entrepreneur who can design technology interventions that can elicit positive prosocial economic behaviors in their underprivileged CRA district. Third, for a city government whose tax base depends largely on property taxes, being able to pay for precision targeted positive engagement that increases quality of life – and thus property values and property taxes – in any neighborhood in their city. Fourth, for a municipal-bond underwriter being able to insure repayment of their bond by investing in exactly the same scenario as the city government, in the example above.

Implications of the Peace Data Standard for academia suggest its adoption can facilitate the following research avenues: rigorous empirical examination of first and second order economic impacts with types and qualities of engagement episodes mediated by different technologies; rapid, large N hypothesis testing for psychological and sociological impacts of technology platforms; deployment of large scale randomized controlled field trials to validate promising hypotheses in many different environments and under many varying conditions; testing and deployment of machine learning to generate automated rapid responses to changing conditions anywhere in the world. As stated earlier in the manuscript, we believe that this data standard will also allow scholars and entrepreneurs to rapidly design, test, and validate Peace Tech interventions that effectively transform these group differences into raw material for sustainable peace (in which mutual benefit is equal to the cost of engagement) and eventually scalable positive peace (in which mutual benefit exceeds the cost of engagement).

## Author Contributions

REG took lead on writing the manuscript and performing the literature review. MN was the source of the idea presented in the paper and contributed to the writing. LLL provided the data presented in Example 3.

## Conflict of Interest Statement

LLL was employed by company SWOOP Analytics. The other authors declare that the research was conducted in the absence of any commercial or financial relationships that could be construed as a potential conflict of interest.

## Footnotes

1 Note that we intentionally using the phrase “mediating technology” rather than “mediated technology” to suggest that, in addition to technology being used as a means of communication (as in the case of “mediated communication”), it can also serve as a virtual stand in for a mediator – an objective third party that assists with conflict resolution. That said, the phrase “mediated technology” also appears in the manuscript and it refers to the more traditional definition.

2 http://gamesforpeace.org

3 http://thepeacefactory.org

4 https://www.facebook.com/israellovesiran/

5 https://www.facebook.com/peace

6 This example pertains to gender, the social construct, rather than biological sex. It was not our intention to exclude people with more fluid, less easily categorizable, gender identities.

7 None of the authors of the present paper have the appropriate academic credentials (e.g., none of us are, for instance, a behavioral economist) so linking economic value to Peace Data is beyond the scope of the present paper. Nonetheless, we think the potential to link financial outcomes is there and are on the lookout for collaborators to help further explore this research direction.

8 https://products.office.com/en-us/yammer/yammer-overview

9 The academic home of the first and second authors of this paper.
